# Application of the Intuitionistic Fuzzy InterCriteria Analysis Method with Triples to a Neural Network Preprocessing Procedure

**DOI:** 10.1155/2017/2157852

**Published:** 2017-08-10

**Authors:** Sotir Sotirov, Vassia Atanassova, Evdokia Sotirova, Lyubka Doukovska, Veselina Bureva, Deyan Mavrov, Jivko Tomov

**Affiliations:** ^1^Laboratory of Intelligent Systems, University “Prof. Dr. Assen Zlatarov”, 1 “Prof. Yakimov” Blvd., 8010 Burgas, Bulgaria; ^2^Department of Bioinformatics and Mathematical Modelling, Institute of Biophysics and Biomedical Engineering, 105 “Acad. G. Bonchev” Str., 1113 Sofia, Bulgaria; ^3^Department of Intelligent Systems, Institute of Information and Communication Technologies, 2 “Acad. G. Bonchev” Str., 1113 Sofia, Bulgaria

## Abstract

The approach of InterCriteria Analysis (ICA) was applied for the aim of reducing the set of variables on the input of a neural network, taking into account the fact that their large number increases the number of neurons in the network, thus making them unusable for hardware implementation. Here, for the first time, with the help of the ICA method, correlations between triples of the input parameters for training of the neural networks were obtained. In this case, we use the approach of ICA for data preprocessing, which may yield reduction of the total time for training the neural networks, hence, the time for the network's processing of data and images.

## 1. Introduction

Working with neural networks presents many difficulties; for example, the number of neurons in the perception of the individual values can be too large, and since a proportionally larger amount of memory and computing power is necessary to train the networks, this would lead to a longer periods for training. Therefore, researchers are forced to look for better methods for training neural networks. Backpropagation is the most applied such method—in it neural networks are trained with uplink (applied on a Multilayer Perceptron). There are, however, many other methods that accelerate the training of neural networks [1–3], by reducing memory usage, which in turn lowers the needed amount of computing power.

In the stage of preprocessing, the data at the input of the neural network can be used as a constant threshold value to distinguish static from dynamic activities, as it was done in [4]. This way, the amount of incidental values due to unforeseen circumstances is reduced.

Another approach is to use a wavelet-based neural network classifier to reduce the power interference in the training of the neural network or randomly stumbled measurements [5]. Here the discrete wavelet transform (DWT) technique is integrated with the neural network to build a classifier.

Particle Swarm Optimization (PSO) is an established method for parameter optimization. It represents a population-based adaptive optimization technique that is influenced by several “strategy parameters.” Choosing reasonable parameter values for PSO is crucial for its convergence behavior and depends on the optimization task. In [6] a method is presented for parameter metaoptimization based on PSO and it is applied to neural network training. The idea of Optimized Particle Swarm Optimization (OPSO) is to optimize the free parameters of PSO by having swarms within a swarm.

When working with neural networks it is essential to reduce the amount of neurons in the hidden layer, which also reduces the number of weight coefficients of the neural network as a whole. This leads to a smaller dimension of the weight matrices, and hence the used amount of memory. An additional consequence from this is the decreased usage of computing power and the shortened training time [7].

Multilayer Perceptrons are often used to model complex relationships between sets of data. The removal of nonessential components of the data can lead to smaller sizes of the neural networks, and, respectively, to lower requirements for the input data. In [8] it is described that this can be achieved by analyzing the common interference of the network outputs, which is caused by distortions in the data that is passed to the neural network's inputs. The attempt to find superfluous data is based on the concept of sensitivity of linear neural networks. In [9] a neural network is developed, in which the outputs of the neurons of part of the layers are not connected to the next layer. The structure thus created is called a “Network in a Network.” In this way part of the inputs of the neural network are reduced, which removes part of the information, and along with it part of the error accumulated during training and data transfer. The improved local connection method given in [9] produces a global collation by fundamental cards in the classification layer. This layer is easier to interpret and less prone to overloading than the traditional fully connected layers.

In this paper, we apply the intuitionistic fuzzy sets-based method of InterCriteria Analysis to reduce the number of input parameters of a Multilayer Perceptron. This will allow the reduction of the weight matrices, as well as the implementation of the neural network in limited hardware, and will save time and resources in training.

The neural network is tested after reducing the data (effectively the number of inputs), so as to obtain an acceptable relation between the input and output values, as well as the average deviation (or match) of the result.

## 2. Presentation of the InterCriteria Analysis

The InterCriteria Analysis (ICA) method is introduced in [10] by Atanassov et al. It can be applied to multiobject multicriteria problems, where measurements according to some of the criteria are slower or more expensive, which results in delaying or raising the cost of the overall process of decision-making. When solving such problems it may be necessary to adopt an approach for reasonable elimination of these criteria, in order to achieve economy and efficiency.

The ICA method is based on two fundamental concepts: intuitionistic fuzzy sets and index matrices. Intuitionistic fuzzy sets were first defined by Atanassov [11–13] as an extension of the concept of fuzzy sets defined by Zadeh [14]. The second concept on which the proposed method relies is the concept of index matrix, a matrix which features two index sets. The theory behind the index matrices is described in [15].

According to the ICA method, a set of objects is evaluated or measured against a set of criteria, and the table with these evaluations is the input for the method. The number of criteria can be reduced by calculating the correlations (differentiated in ICA to: positive consonance, negative consonance, and dissonance) in each pair of criteria in the form of intuitionistic fuzzy pairs of values, that is, a pair of numbers in the interval [0,1], whose sum is also a number in this interval. If some (slow, expensive, etc.) criteria exhibit positive consonance with some of the rest of the criteria (that are faster, cheaper, etc.), and this degree of consonance is considered high enough with respect to some predefined thresholds, with this degree of precision the decision maker may decide to omit them in the further decision-making process. The higher the number of objects involved in the measurement, the more precise the evaluation of the intercriteria consonances (correlations). This makes the approach completely data-driven and ongoing approbations over various application problems and datasets are helping us better perceive its reliability and practical applicability.

Let us consider a number of *C*_*q*_ criteria, *q* = 1,…, *n*, and a number of *O*_*p*_ objects, *p* = 1,…, *m*; that is, we use the following sets: a set of criteria *C*_*q*_ = {*C*_1_,…, *C*_*n*_} and a set of objects *O*_*p*_ = {*O*_1_,…, *O*_*m*_}.

We obtain an index matrix* M* that contains two sets of indices, one for rows and another for columns. For every* p*,* q* (1 ≤ *p* ≤ *m*, 1 ≤ *q* ≤ *n*), *O*_*p*_ in an evaluated object, *C*_*q*_ is an evaluation criterion, and *a*_*O*_*p*_,*C*_*q*__ is the evaluation of the* p*th object against the* q*th criterion, defined as a real number or another object that is comparable according to a relation *R* with all the other elements of the index matrix* M.*

(1) $$\begin{array}{c} M=\begin{array}{cccccccc} & C_{1} & \cdots & C_{k} & \cdots & C_{l} & \cdots & C_{n}\\ O_{1} & a_{O_{1},C_{1}} & \cdots & a_{O_{1},C_{k}} & \cdots & a_{O_{1},C_{l}} & \cdots & a_{O_{1},C_{n}}\\ \cdots & \cdots & \cdots & \cdots & \cdots & \cdots & \cdots & \cdots \\ O_{i} & a_{O_{i},C_{1}} & \cdots & a_{O_{i},C_{k}} & \cdots & a_{O_{i},C_{l}} & \cdots & a_{O_{i},C_{n}}\\ \cdots & \cdots & \cdots & \cdots & \cdots & \cdots & \cdots & \cdots \\ O_{j} & a_{O_{j},C_{1}} & \cdots & a_{O_{j},C_{k}} & \cdots & a_{O_{j},C_{l}} & \cdots & a_{O_{j},C_{n}}\\ \cdots & \cdots & \cdots & \cdots & \cdots & \cdots & \cdots & \cdots \\ O_{m} & a_{O_{m},C_{1}} & & a_{O_{m},C_{k}} & & a_{O_{m},C_{l}} & \cdots & a_{O_{m},C_{n}}. \end{array} \end{array}$$

The next step is to apply the InterCriteria Analysis for calculating the evaluations. The result is a new index matrix *M*^*∗*^ with intuitionistic fuzzy pairs 〈*μ*_*C*_*k*_,*C*_*l*__, *ν*_*C*_*k*_,*C*_*l*__〉 that represents an intuitionistic fuzzy evaluation of the relations between every pair of criteria* C*_*k*_ and* C*_*l*_. In this way the index matrix* M* that relates the evaluated objects with the evaluating criteria can be transformed to another index matrix *M*^*∗*^ that gives the relations among the criteria:

(2) $$\begin{array}{c} M^{*}=\begin{array}{cccc} & C_{1} & \cdots & C_{n}\\ C_{1} & \langle \mu_{C_{1},C_{1}},\nu_{C_{1},C_{1}}\rangle & \cdots & \langle \mu_{C_{1},C_{n}},\nu_{C_{1},C_{n}}\rangle \\ \cdots & \cdots & \cdots & \cdots \\ C_{n} & \langle \mu_{C_{q},C_{1}},\nu_{C_{q},C_{1}}\rangle & \cdots & \langle \mu_{C_{n},C_{n}},\nu_{C_{n},C_{n}}\rangle \end{array} \end{array}$$

The last step of the algorithm is to determine the degrees of correlation between groups of indicators depending of the chosen thresholds for *μ* and *ν* from the user. The correlations between the criteria are called “positive consonance,” “negative consonance,” or “dissonance.” Here we use one of the possible approaches to defining these thresholds, namely, the scale shown in Box 1 [16].

## 3. InterCriteria Analysis with Triples

The algorithm for identifying intercriteria triples is introduced in [17] by Atanassova et al.

**Step 1 .**

Starting from the input dataset of *m* objects measured against *n* criteria, we calculate the total number of *n*(*n* − 1)/2 intuitionistic fuzzy pairs standing for the intercriteria consonances and plot these pairs as points onto the intuitionistic fuzzy triangle. Instead of maintaining a pair of two numbers for each pair of criteria *C*_*i*_-*C*_*j*_, namely, 〈*μ*_*ij*_, *ν*_*ij*_〉 we calculate (see [18]) for each pair the number *d*_*ij*_:

(3) $$\begin{array}{c} d_{ij}=\sqrt{{\left(1-\mu_{ij}\right)}^{2}+\nu_{ij}^{2}} \end{array}$$

giving its distance from the (1; 0) point, that is, the image of the complete Truth onto the intuitionistic fuzzy triangle. Our aim is to identify top-down all the *n*(*n* − 1)/2 calculated values that are closest to the (1; 0) and, at the same time, closest to each other; hence we sort them in ascending order by their distance to (1; 0); see the example in Table 2.

**Step 2 .**

**Figure 1:**
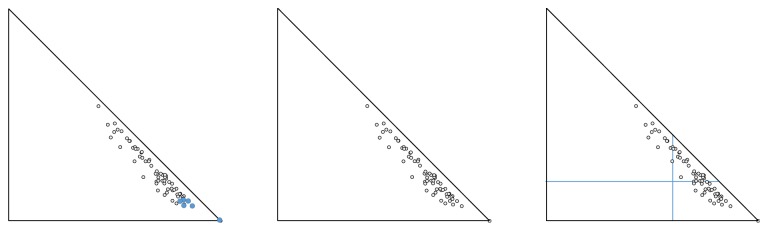
Three alternatives for constructing the subset Σ [17].

Let us denote with Σ the subset of the closest to (1; 0) triples of criteria. The way we construct the subset Σ may slightly differ per user preference or external requirement, with at least three possible alternatives, as listed below (see Figure 1):

(2.1) Select top *p* or top* q*% of the *n*(*n* − 1)/2 ICA pairs (predefined number of elements of the subset Σ).
(2.2) Select all ICA pairs whose corresponding points are within a given radius *r* from the (1; 0) point.
(2.3) Select all ICA pairs whose corresponding points fall within the trapezoid formed between the abscissa, the hypotenuse, and the two lines corresponding to *y* = *α* and *x* = *β* for two predefined numbers *α*, *β* ∈ [0; 1].

**Step 3 .**

Check if there are triples of criteria, each pair of which corresponds to a point, belonging to the subset Σ. If no, then no triples of criteria conform with the stipulated requirements. However, if triples are to be found, then we extend the subset Σ accordingly, by either taking a larger number *p* or *q* (Substep (2.1)), or a larger radius *r* (Substep (2.2)), or smaller *α* and/or larger *β* (Substep (2.3)). If now the subset Σ contains triples of criteria that simultaneously fulfil the requirements, then go to Step 4.

**Step 4 .**

We start top-down with the first pair of criteria, let it be* C*_*i*_-*C*_*j*_, that is, the pair with the smallest *d*_*ij*_, thus ensuring maximal proximity of the corresponding point, say, *P*_*ij*_, to (1; 0) point. We may pick the third criterion in the triple either as *C*_*k*_ which is the next highest correlating criterion with *C*_*l*_, that is, *P*_*ik*_ with *d*_*ik*_ (>*d*_*ij*_), or as *C*_*i*_ which is the next highest correlating criterion with *C*_*j*_, that is, *P*_*jl*_ with *d*_*jl*_ (>*d*_*ij*_, noting that it is possible to have *d*_*ik*_ = *d*_*jl*_). Then, we check the distances to (1; 0) of the respective third points *P*_*jk*_ and *P*_*il*_, taking that triple of criteria* C*_*i*_-*C*_*j*_-*C*_*k*_ or* C*_*i*_-*C*_*j*_-*C*_*l*_ that has the

(4) $$\begin{array}{c} \min\left(\;d_{ij}+d_{ik}+d_{jk},d_{ij}+d_{il}+d_{jl}\;\right). \end{array}$$

Then for each triple of criteria* C*_*i*_-*C*_*j*_-*C*_*x*_ (where *x* ∈ {*k*, *l*}), we calculate the median point of the so formed triangle, which is a point plotted in the intuitionistic fuzzy triangle with coordinates:

(5) $$\begin{array}{c} \langle \;\tilde{\mu },\tilde{\nu }\;\rangle =\langle \;\frac{\mu_{ij}+\mu_{jx}+\mu_{xi}}{3},\frac{\nu_{ij}+\nu_{jx}+\nu_{xi}}{3}\;\rangle . \end{array}$$

This pair gives us the level of $\langle \tilde{\mu },\tilde{\nu }\rangle$-consonance of the whole triple. Repeat Step 4 until the number of the triples in the subset Σ is exhausted.

## 4. Artificial Neural Networks

**Figure 2:**
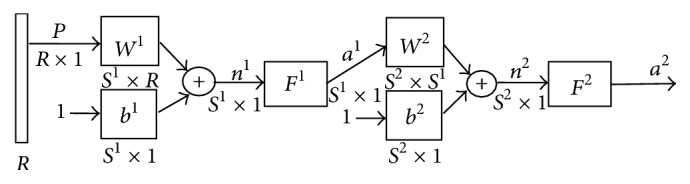
Abbreviated notation of a classical Multilayer Perceptron.

The artificial neural networks [4, 19] are one of the tools that can be used for object recognition and identification. In the first step, it has to be learned and after that we can use for the recognitions and for predictions of the properties of the materials.  Figure 2 shows in abbreviated notation of a classic two-layered neural network.

In the two-layered neural networks, one layer's exits become entries for the next one. The equations describing this operation are

(6) $$\begin{array}{c} a^{2}=f^{2}\left(\;w^{2}f^{1}\left(\;w^{1}p+b^{1}\;\right)+b^{2}\;\right), \end{array}$$

where

- *a*^*m*^ is the exit of the* m*th layer of the neural network for *m* = 1,2;
- *w*^*m*^ is a matrix of the weight coefficients of the each of the entries of the *m*th layer;
- *b* is the neuron's entry bias;
- *f*^1^ is the transfer function of the 1st layer;
- *f*^2^ is the transfer function of the 2nd layer.

The neuron in the first layer receives *p* outside entries. The neurons' exits from the last layer determine the neural network's exits as *a*.

The “backpropagation” algorithm [20] is used for learning the neural networks. When the multilayer neural network is trained, usually the available data has to be divided into three subsets. The first subset, named “Training set,” is used for computing the gradient and updating the network weights and biases. The second subset is named “Validation set.” The error of the validation set is monitored during the training process. The validation error normally decreases during the initial phase of training, as does the training set error. Sometimes, when the network begins to overfit the data, the error of the validation set typically begins to rise. When the validation error increases for a specified number of iterations, the training stops and the weights and biases at the minimum of the validation error are returned [4]. The last subset is named “test set.” The sum of these three sets has to be 100% of the learning couples.

For this investigation we use MATLAB and neural network structure 8:45:1 (8 inputs, 45 neurons in hidden layer, and one output) (Figure 2). The numbers of the weight coefficients are 9 × 45 = 405.

The proposed method is focused on removing part of the number of neurons (and weight coefficients) and thus does not reduce the average deviation of the samples, used for the learning testing and validating the neural network.

## 5. Testing

We consider a number of *C*_*q*_ criteria, *q* = 1,…, *n*, and a number of *O*_*p*_ measurements of cetane number of crude oil, *p* = 1,…, *m*; that is, we use the following sets: a set of group of criteria *C*_*q*_ = {*C*_1_,…, *C*_*n*_} and a set of measurements of cetane number *O*_*p*_ = {*O*_1_,…, *O*_*m*_}.

The ICA method was applied to the 140 crude oil probes, measured against 8 criteria as listed below:

- density at 15°C g/cm^3^;
- 10% (v/v) ASTM D86 distillation, °C;
- 50% (v/v) ASTM D86 distillation, °C;
- 90% (v/v) ASTM D86 distillation, °C;
- refractive index at 20°C;
- H_2_ content, % (m/m);
- aniline point, °C;
- molecular weight g/mol.

So we work with a 140 × 8 table, and a software application that implements the ICA algorithm returns the results in the form of two index matrices (see Tables 1 and 2), containing, respectively, the membership and the nonmembership parts of the intuitionistic fuzzy correlations detected between each pair of criteria (28 pairs). The values in the matrix are colored in red-yellow-green color scale for the varying degrees of consonance and dissonance from green (highest values) to yellow. Naturally, each criterion best correlates with itself, which gives the respective intuitionistic fuzzy pairs 〈1; 0〉, or 1s and 0s, along the main diagonals of Tables 1 and 2.

In Table 3 the relations between the pairs of criteria obtained by applying the ICA method are shown.

The calculated distance *d*_*ij*_ for each pair of criteria* C*_*i*_-*C*_*j*_ from the (1; 0) point in the intuitionistic fuzzy triangle is shown in Table 4 (note that *d*_*ij*_ ∈ [0, √2]).

The next step is to choose the pair* C*_*i*_*-C*_*j*_ with the smallest *d*_*ij*_, thus ensuring maximal proximity of the corresponding point to (1; 0) point. We pick the third criterion in the triple either as *C*_*k*_ that is the next highest correlating criterion with *C*_*i*_, or as *C*_*l*_ that is the next highest correlating criterion with *C*_*j*_, taking that triple of criteria* C*_*i*_*-C*_*j*_*-C*_*k*_ or* C*_*i*_*-C*_*j*_*-C*_*l*_ that has the min⁡(*d*_*ij*_ + *d*_*ik*_ + *d*_*jk*_, *d*_*ij*_ + *d*_*il*_ + *d*_*jl*_). In Table 5 the pairs of criteria* C*_*i*_*-C*_*j*_ in “strong positive consonance,” “positive consonance,” and “weak positive consonance” are shown.

On the input of the neural network we put the experimental data for obtaining cetane number of crude oil. Testing is done as at the first step; all the measurements of the 140 crude oil probes against the 8 criteria are analyzed in order to make a comparison of the obtained results thereafter. For this comparison to be possible, the predefined weight coefficients and offsets that are normally random values between −1 and 1 are now established and are the same in all studies with coefficients 1.

For the learning process, we set the following parameters: performance (MSE) = 0.00001; validation check = 25. The input vector is divided into three different parts: training (70/100); validation (15/100); and testing (15/100). For target we use the cetane number ASTM D613.

At the first step of the testing process, we use all the 8 criteria listed above, in order to train the neural network. After the training process all input values are simulated by the neural network.

The average deviation of the all 140 samples is 1,8134. The coefficient *R* (regression *R* values measure the correlation between outputs and targets) obtained from the MATLAB program is 0.97434 (see Table 6).

At the next step of the testing process, we make a fork and try independently to remove one of the columns and experiment with data from the remaining seven columns. We compare the results in the next section, “Discussion.” First, we make a reduction of column 1 (based on Table 5) and put the data on the input of the neural network.

After the training process all input values are simulated. The average deviation of all the 140 samples is 1.63 and the coefficient *R* is 0.9772.

At the next step, we alternatively perform reduction of column 3 (according to Table 5), and put the data on the input of the neural network.

After the training process all input values are simulated. The average deviation of the all 140 samples is 1.8525 and the coefficient *R* is 0.97256. After that we can proceed with columns 5, 2, 8, and 4.

Now, at the next step, we proceed with feeding the neural network with 6 inputs, with the reduction of both columns, 3 and 5, according to the data from Table 5. The average deviation of all the 140 samples is 1.7644 and the coefficient *R* is 0.97089. In the same way we can reduce the inputs: 1 and 5, 1 and 3, 2 and 3, 3 and 8, 3 and 4, and 4 and 8, simultaneously.

At the next step, we reduce the number of inputs with one more, that is, we put on the input of the neural network experimental data from 5 inputs, with removed columns 1, 3, and 5. The average deviation of all the 140 samples is 1.857 and the coefficient *R* is 0.97208 (see Table 6). In the same way are removed the parameters 2, 3, and 8 and 3, 4, and 8.

Finally, we experiment with the reduction of the fourth column, feeding the neural network with only 4 inputs. After the reduced columns 1, 2, and 4, the fourth reduced column is column 5. After the simulation the average deviation of the all 140 samples is 2.19 and the coefficient *R* obtained from the MATLAB program is 0.95927.

## 6. Discussion

In support of the method, Tables 6, 7, and 8 present the correlation coefficients between the different criteria. The tables also present the maximal values of the coefficient sums per criteria. In the last column, the triples of selected criteria are given, as sorted in the descending way by the* correlation coefficient C*_*i*_*-C*_*j*_.

In Table 9 compilations between ICA approach and correlation analysis according to Pearson, Kendall, and Spearman are shown.

The selected pairs, based on the four methods, are identical in the first row. In the second row three of the methods yield identical results (ICA, Kendall, and Spearman), and the only difference is in the selected criteria as calculated by the Pearson method. In the third row, the situation is the same. Here the triples are the same with precision of ordering. Only the triple of correlation criteria calculated by the* Pearson* method is different. In the fourth row, the triples are quite similar. The triples calculated by* ICA and Pearson* are identical. The triple determined by* Kendall* correlation coincides with the first row of the table. The last triple, defined by the* Spearman* correlation, coincides with the second and third row of the triples defined by the correlation analyses of* ICA, Pearson*,* and Spearman*.

So far, such a detailed comparison between the four methods has been conducted over medical [21, 22] and petrochemical [23] data. It was observed that considerable divergence of the ICA results from the results obtained by the rest of the methods is only found when the input data contain mistakes, as a result of misplacing the decimal point with at least one position to the left or to the right. We anticipate in the future a theoretical research for checking the validity of this practical observation. If it proves to be true, then ICA, together with the rest three types of analysis, will turn into a criterion for data correctness.

As we stated above, reducing the number of input parameters of a classical neural network leads to reduction of the weight matrices, resulting in implementation of the neural network in limited hardware and saving time and resources in training. For this aim, we use the intuitionistic fuzzy sets-based approach of InterCriteria Analysis (ICA), which gives dependencies between the criteria and thus helps us reduce the number of highly correlating input parameters, yet keeping high enough the level of precision.

Table 10 summarizes the most significant parameters of the process of testing the neural network with different numbers of inputs, gradually reducing the number in order to discover optimal results. These process parameters are the NN-specific parameters “average deviation,” “regression coefficient* R*,” and “number of the weight coefficients.”

The average deviation when we use 8 input vectors is 1.8134 with number of weight coefficients 405. By reducing the number of the inputs the number of weight coefficients is also decreased which theoretically is supposed to reduce the matching coefficient. In this case the removal of column 1 (and therefore one input is removed) causes further decreasing the average deviation of 1.6327. The additional information (without column 5) used for training the neural network is very little, and the total Mean Square Error is less. The result is better compared to the formerly used attempt by training the neural network with 8 data columns.

When we use 7 columns (and 7 inputs of neural networks) excluding some of the columns gives better result than the previous one. This shows that, while maintaining the number of weight coefficients and reducing the maximal membership in the intercriteria IF pairs, the neural network receives an additional small amount of information which it uses for further learning.

Best results (average deviation = 1.5716) are obtained by removing the two columns (6 inputs without inputs 1 and 3) with the greatest membership components of the respective* d*.

In this case, the effect of reducing the number of weight coefficients from 360 to 315 and the corresponding MSE is greater than the effect of the two columns.

The use of 5 columns (without columns 1, 3, and 5) leads to a result which is less than the previous, that is, 1.857. This shows that with reducing the number of weight coefficients (and the total MSE) and the information at the input of the neural network a small amount of information is lost with which the network is trained. As a result, the overall accuracy of the neural network is decreased.

The worst results (average deviation = 2.217) are obtained in the lowest number of columns—4. In this case, columns 1, 2, 4, and 5 are removed. Although the number of weight coefficients here is the smallest, the information that is used for training the neural network is less informative.

## 7. Conclusion

In the paper we apply the newest leg of theoretical research on InterCriteria Analysis to a dataset with the measurements of 140 probes of crude oil against 8 physicochemical criteria. On the first step we put all data from these measurements in the input of a classical neural network. After performing ICA analysis of the pairwise intercriteria correlations, we apply the recently developed method for identification of intercriteria triples in attempt to reduce the inputs of the neural network, without significant loss of precision. This leads to a reduction of the weight matrices, thus allowing implementation of the neural network on limited hardware and saving time and resources in training.

Very important aspect of the testing of the neural network after reducing some of the data (resp., the number of inputs) is to obtain an acceptable correlation between the input and output values, as well as the average deviation (or match) of the result.

## Figures and Tables

**Box 1:**
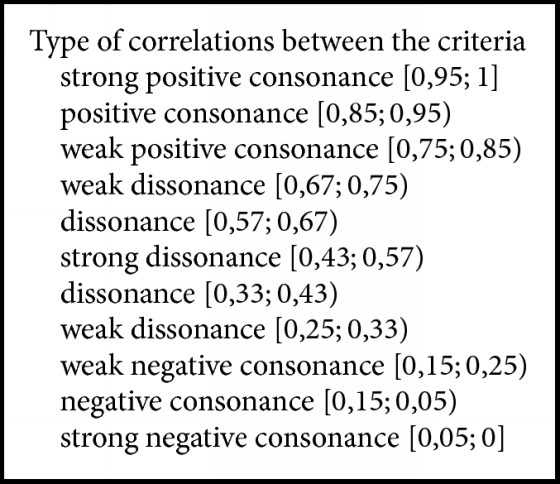
Type of correlations.

**Table 1:** Membership parts of the IF pairs, giving the InterCriteria correlations.

*µ*	(I)	(II)	(III)	(IV)	(V)	(VI)	(VII)	(VIII)
(I)	1	0.699	0.770	0.658	0.956	0.176	0.446	0.703
(II)	0.699	1	0.787	0.597	0.676	0.408	0.640	0.775
(III)	0.770	0.787	1	0.777	0.728	0.395	0.665	0.922
(IV)	0.658	0.597	0.777	1	0.627	0.468	0.674	0.771
(V)	0.956	0.676	0.728	0.627	1	0.134	0.404	0.661
(VI)	0.176	0.408	0.395	0.468	0.134	1	0.730	0.473
(VII)	0.446	0.640	0.665	0.674	0.404	0.730	1	0.743
(VIII)	0.703	0.775	0.922	0.771	0.661	0.473	0.743	1

**Table 2:** Nonmembership parts of the IF pairs, giving the InterCriteria relations.

*ν*	(I)	(II)	(III)	(IV)	(V)	(VI)	(VII)	(VIII)
(I)	0	0.288	0.217	0.326	0.042	0.822	0.552	0.295
(II)	0.288	0	0.204	0.391	0.312	0.580	0.348	0.213
(III)	0.217	0.204	0	0.212	0.261	0.595	0.325	0.068
(IV)	0.326	0.391	0.212	0	0.359	0.518	0.312	0.215
(V)	0.042	0.312	0.261	0.359	0	0.866	0.596	0.339
(VI)	0.822	0.580	0.595	0.518	0.866	0	0.270	0.527
(VII)	0.552	0.348	0.325	0.312	0.596	0.270	0	0.257
(VIII)	0.295	0.213	0.068	0.215	0.339	0.527	0.257	0

**Table 3:** Correlations between the pairs of criteria.

Type of InterCriteria Relation	Pairs of criteria
Strong positive consonance [0.95; 1]	(I-V)
Positive consonance [0.85; 0.95)	(III-VIII)
Weak positive consonance [0.75; 0.85)	(II-III, III-IV, II-VIII, IV-VIII, I-III)
Weak dissonance [0.67; 0.75)	(VII-VIII, III-V, VI-VII, I-II, I-VIII, II-V, IV-VII)
Dissonance [0.57; 0.67)	(III-VII, I-IV, V-VIII, II-VII, IV-V, II-IV)
Strong dissonance [0.43; 0.57)	(IV-VI, VI-VIII, I-VII)
Dissonance [0.33; 0.43)	(II-VI, V-VII, III-VI)
Weak dissonance [0.25; 0.33)	0
Weak negative consonance [0.15; 0.25)	(I-VI)
Negative consonance [0.15; 0.05)	(V-VI)
Strong negative consonance [0.05; 0]	0

**Table 4:** Distance *d*_*ij*_ for each pair of criteria *C*_*i*_*-C*_*j*_.

*d*	(I)	(II)	(III)	(IV)	(V)	(VI)	(VII)	(VIII)
(I)	0	0.416	0.316	0.473	0.061	1.165	0.783	0.419
(II)	0.416	0	0.295	0.561	0.450	0.829	0.501	0.310
(III)	0.316	0.295	0	0.307	0.377	0.849	0.467	0.104
(IV)	0.473	0.561	0.307	0	0.518	0.742	0.452	0.314
(V)	0.061	0.450	0.377	0.518	0	1.225	0.843	0.480
(VI)	1.165	0.829	0.849	0.742	1.225	0	0.382	0.745
(VII)	0.783	0.501	0.467	0.452	0.843	0.382	0	0.363
(VIII)	0.419	0.310	0.104	0.314	0.480	0.745	0.363	0

**Table 5:** Distance *d*_*ij*_ for pair of criteria *C*_*i*_*-C*_*j*_ in positive consonance.

*C*_*i*_	*C*_*j*_	*m*_*ij*_	*d*_*ij*_	*C*_*k*_	*m*_*ik*_	*d*_*ik*_	*d*_*jk*_	*C*_*l*_	*m*_*jl*_	*d*_*il*_	*d*_*jl*_	min(*d*_*ij*_ + *d*_*ik*_ + *d*_*jk*_, *d*_*ij*_ + *d*_*il*_ + *d*_*jl*_)	Chosen triple of criteria	$\langle \tilde{\mu },\tilde{\nu }\rangle$

(I)	(V)	0.956	0.061	(III)	0.770	0.319	0.377	(III)	0.728	0.319	0.377	0.756	*C*_(I)_-*C*_(V)_-*C*_(III)_	〈0.818; 0.173〉
(III)	(VIII)	0.922	0.104	(II)	0.787	0.295	0.310	(II)	0.775	0.295	0.310	0.709	*C*_(III)_-*C*_(VIII)_-*C*_(II)_	〈0.828; 0.162〉
(II)	(III)	0.787	0.295	(VIII)	0.775	0.310	0.104	(IV)	0.777	0.561	0.307	0.709	*C*_(II)_-*C*_(III)_-*C*_(VIII)_	〈0.828; 0.162〉
(III)	(IV)	0.777	0.307	(I)	0.770	0.319	0.473	(VIII)	0.771	0.104	0.314	0.725	*C*_(III)_-*C*_(IV)_-*C*_(VIII)_	〈0.823; 0.165〉
(II)	(VIII)	0.775	0.310	(I)	0.699	0.416	0.418	(IV)	0.771	0.561	0.314	1.144	*C*_(II)_-*C*_(VIII)_-*C*_(I)_	〈0.726; 0.265〉
(IV)	(VIII)	0.771	0.314	(VII)	0.674	0.452	0.363	(VII)	0.743	0.452	0.363	1.129	*C*_(IV)_-*C*_(VIII)_-*C*_(VII)_	〈0.729; 0.261〉
(I)	(III)	0.770	0.316	(VIII)	0.703	0.418	0.104	(V)	0.728	0.061	0.377	0.753	*C*_(I)_-*C*_(III)_-*C*_(V)_	〈0.818; 0.173〉

**Table 6:** Correlation coefficients for pair of criteria *C*_*i*_*-C*_*j*_ according to Pearson.

*C*_*i*_	*C*_*j*_	Correlation coefficient *C*_*i*_-*C*_*j*_	*C*_*k*_	Correlation coefficient *C*_*i*_-*C*_*k*_	*C*_*l*_	Correlation coefficient *C*_*j*_*-C*_*l*_	max(correlation coefficient *C*_*i*_*-C*_*j*_+ correlation coefficient *C*_*i*_*-C*_*k*_; correlation coefficient *C*_*i*_*-C*_*j*_+ correlation coefficient *C*_*j*_*-C*_*l*_)	Chosen triple of criteria
(I)	(V)	0,989	(III)	0,616	(III)	0,495	1,605	(I-V-III)
(III)	(VIII)	0,971	(IV)	0,819	(II)	0,797	1,789	(III-VIII-IV)
(VI)	(VII)	0,831	(VIII)	0,024	(VIII)	0,576	1,406	(VI-VII-VIII)
(III)	(IV)	0,819	(VIII)	0,971	(VIII)	0,796	1,789	(III-IV-VIII)

**Table 7:** Correlation coefficients for pair of criteria *C*_*i*_*-C*_*j*_ according to Kendall.

*C*_*i*_	*C*_*j*_	Correlation coefficient *C*_*i*_-*C*_*j*_	*C*_*k*_	Correlation coefficient *C*_*i*_-*C*_*k*_	*C*_*l*_	Correlation coefficient *C*_*j*_-*C*_*l*_	max(correlation coefficient *C*_*i*_-*C*_*j*_+ correlation coefficient *C*_*i*_-*C*_*k*_; correlation coefficient *C*_*i*_-*C*_*j*_+ correlation coefficient *C*_*j*_-*C*_*l*_)	Chosen triple of criteria
(I)	(V)	0,915	(III)	0,557	(III)	0,470	1,472	(I-V-III)
(III)	(VIII)	0,858	(II)	0,582	(II)	0,566	1,440	(III-VIII-II)
(II)	(III)	0,582	(VIII)	0,566	(VIII)	0,566	1,147	(II-III-VIII)
(I)	(III)	0,557	(V)	0,915	(VIII)	0,858	1,472	(I-III-V)

**Table 8:** Correlation coefficients for pair of criteria *C*_*i*_-*C*_*j*_ according to Spearman.

*C*_*i*_	*C*_*j*_	Correlation coefficient *C*_*i*_-*C*_*j*_	*C*_*k*_	Correlation coefficient *C*_*i*_-*C*_*k*_	*C*_*l*_	Correlation coefficient *C*_*j*_-*C*_*l*_	max(correlation coefficient *C*_*i*_-*C*_*j*_+ correlation coefficient *C*_*i*_-*C*_*k*_; correlation coefficient *C*_*i*_-*C*_*j*_+ correlation coefficient *C*_*j*_-*C*_*l*_)	Chosen triple of criteria
(I)	(V)	0,988	(III)	0,728	(III)	0,641	1,716	(I-V-III)
(III)	(VIII)	0,962	(II)	0,762	(II)	0,753	1,724	(III-VIII-II)
(II)	(III)	0,762	(VIII)	0,753	(VIII)	0,962	1,724	(II-III-VIII)
(II)	(VIII)	0,753	(III)	0,762	(III)	0,962	1,715	(II-VIII-III)

**Table 9:** 

	ICA	Pearson	Kendall	Spearman
(1)	(I-V-III)	(I-V-III)	(I-V-III)	(I-V-III)
(2)	(III-VIII-II)	(III-VIII-IV)	(III-VIII-II)	(III-VIII-II)
(3)	(II-III-VIII)	(VI-VII-VIII)	(II-III-VIII)	(II-III-VIII)
(4)	(III-IV-VIII)	(III-IV-VIII)	(I-III-V)	(II-VIII-III)

**Table 10:** Table of comparison.

Number of inputs	Average deviation	Regression coefficient *R*	Number of the weight coefficients
8 inputs	1.8134	0.97434	405
7 inputs without input 1	1.6327	0.9772	360
7 inputs without input 3	1.8525	0.97256	360
7 inputs without input 5	1.6903	0.9734	360
7 inputs without input 2	2.1142	0.96511	360
7 inputs without input 8	1.7735	0.97511	360
7 inputs without input 4	1.9913	0.96932	360
6 inputs without inputs 3, 5	1.7644	0.97089	315
6 inputs without inputs 1, 5	1.8759	0.97289	315
6 inputs without inputs 1, 3	1.5716	0.97881	315
6 inputs without inputs 2, 3	2.0716	0.96581	315
6 inputs without inputs 3, 8	1.9767	0.97213	315
6 inputs without inputs 3, 4	1.9792	0.97163	315
6 inputs without inputs 4, 8	2.0174	0.96959	315
5 inputs without inputs 1, 3, 5	1.857	0.97209	270
5 inputs without inputs 2,3, 8	2.0399	0.96713	270
5 inputs without inputs 3, 4, 8	2.0283	0.96695	270
4 inputs without inputs 1, 2, 4, 5	2.217	0.95858	225
4 inputs without inputs 2, 3, 4, 8	2.1989	0.95927	225
